# Fisher Information Based Meteorological Factors Introduction and Features Selection for Short-Term Load Forecasting

**DOI:** 10.3390/e20030184

**Published:** 2018-03-09

**Authors:** Shuping Cai, Lin Liu, Huachen Sun, Jing Yan

**Affiliations:** 1 College of Electrical Information and Engineering, Jiangsu University, Zhenjiang 212013, China; 2 Key Laboratory of Facility Agriculture Measurement and Control Technology and Equipment of Machinery Industry, Jiangsu University, Zhenjiang 212013, China

**Keywords:** short-term load forecasting, weather factors, feature selection, Fisher information

## Abstract

Weather information is an important factor in short-term load forecasting (STLF). However, for a long time, more importance has always been attached to forecasting models instead of other processes such as the introduction of weather factors or feature selection for STLF. The main aim of this paper is to develop a novel methodology based on Fisher information for meteorological variables introduction and variable selection in STLF. Fisher information computation for one-dimensional and multidimensional weather variables is first described, and then the introduction of meteorological factors and variables selection for STLF models are discussed in detail. On this basis, different forecasting models with the proposed methodology are established. The proposed methodology is implemented on real data obtained from Electric Power Utility of Zhenjiang, Jiangsu Province, in southeast China. The results show the advantages of the proposed methodology in comparison with other traditional ones regarding prediction accuracy, and it has very good practical significance. Therefore, it can be used as a unified method for introducing weather variables into STLF models, and selecting their features.

## 1. Introduction

Short-term load forecasting (STLF) plays an important role on ensuring power system security and economic operation [1], and its prediction accuracy is influenced by many interdependent factors. Of all these factors, meteorological factors are the dominant exogenous factors that affects STLF [2,3,4]. More recently, meteorological sensitive load demands such as air conditioning, space heating, agricultural irrigation, etc., increasingly grow, and the influence of weather conditions on electrical demands is further intensified, which makes their relation more complicated. Whether weather factors are properly considered has a significant impact on the prediction accuracy of STLF, while even a small improvement in prediction accuracy means big cost savings and also a great contribution to the environment in which we live [5]. Meanwhile, with the wide deployment of real-time monitoring devices in several 220- and 110-kV substations across the forecasted area, it becomes possible to gather real-time weather data such as temperature, humidity, rainfall, wind speed, etc. [6]. In this context, it would be of immense value to develop methods introducing meteorological variables into the forecasting models to improve prediction accuracy and boost prediction speed.

Numerous studies have shown that, of all meteorological factors, temperature has the most remarkable effect on electrical demands [7,8]. Humidity also has a major effect on the comfort or discomfort felt, because it affects the amount of heat that the human body rejects through evaporation [9,10]. Other factors that have an impact on load demand behavior are the wind speed and cloud cover. Rainfall influences electrical demand rather indirectly by affecting other weather variables [11]. Meteorological variables could be introduced into the forecasting models in a primitive form or in a derived form created by combining two or few of them, namely, Meteorological Composite Index (MCI), such as Temperature–humidity index (THI), Comfort Index of Human Body (CIHB), etc. [12]. The MCIs are more popular among conventional models, especially temperature derivatives [13,14]. The MCIs created by mixing different variables such as temperature, humidity, and wind speed are also popular among artificial intelligence models. These MCIs include: Heat Index (HI), introduced by USA National Weather Service [15], tries to represent perception of temperature by a human depending on relative humidity, namely, human-perceived equivalent temperature [16]. Humidex, proposed by Canadian meteorologists Masterton and Richardson in 1979 [17], is another parameter calculated using temperature and humidity. It was utilized in similar day-based wavelet neural network (NN) models [18], and implied models using the MCI performed better than ones using primitives. Temperature–humidity index (THI) is an analysis index used frequently by forecasting models [19]. Through this, both temperature and humidity are introduced into load prediction, which was employed in some models [11,20], and showed its effectiveness. Real sense of temperature is another type of meteorological parameter integrating together temperature, relative humidity and wind speed in an attempt to capture coupling effect of these factors on human perception. A backpropagation based NN model used it as one of its inputs to forecast load demands, and achieved better results [21]. Comfort Index of Human Body (CIHB) is a measure of synthesized effect combining temperature, relative humidity and wind speed. It is an indicator depicting human body’s comfortable level in atmospheric environment [22]. An improved Elman neural network model utilized it as one of the inputs, and compared it with another one using temperature only. As a result, better prediction accuracy was acquired with the former solution [23]. Enthalpy latent days (ELD) is a parameter depicting the influence of humidity on summer load demand for cooling and air-conditioning [24]. It can be expounded as an amount of energy necessary to decreasing indoor humidity to an acceptable level without reducing the indoor temperature. Wind-chill index (WCI) is an index aiming to capture cooling effect of the wind blowing while temperature is below threshold of 50 °F (10 °C) [25]. The WCI was selected as an input to wavelet neural network to correct similar-day based forecasting model, and showed an improvement of the accuracy [18,26]. Cooling power of the wind (CP) was developed by Taylor and Buizza [27], which is a nonlinear function of wind speed and average temperature trying to capture chill induced by gusts of wind.

All the aforementioned MCIs have greatly enriched the approaches of introduction of weather factors into STLF and lay a firm foundation for the improvement of prediction accuracy in STLF. All these approaches introducing meteorological variables into STLF models, however, have some intrinsic limitations due to the complexity of the problems involved. Current load demand is affected not only by the temperature of the current moment but also by the previous one because of the accumulative effect of temperature (AET) [19]. Although Li and Fang et al. [1,28] presented their respective corrected models by taking AET into account, a cumulative effect coefficient based on the models must be adjusted frequently over time. Jiang et al. [29] acquired a quantitative calculation formula for AET by means of curve fitting algorithm, but a proportion coefficient concerning contribution of both continuous number of high temperature days and the highest daily temperature to AET varied over different regions. On the other hand, all the MCIs above involve only the features of daily meteorological factors (Max, Mean, and Min) as their effects on electrical demand. The investigation by Callaway et al. [30] showed that using a simple daily average temperature cannot provide good representations of weather, therefore, cannot obtain a higher prediction accuracy of STLF. Although Kang et al. [31] developed a NN model based on real-time meteorological factors for STLF, the number of input variables grows dramatically, which would inevitably lead to an increase in computational costs. Hence, it is more important to investigate pointwise influence of meteorological factors on the load demands to establish load forecasting models point by point under real-time weather information circumstances.

Information theory provides a new way of introducing weather variables into STFL models. The reason is that essentially any type of data or model can be converted to information regardless of disciplinary origin [32]. Zhu et al. [33] described STLF as a process of information decision-making, and presented a combined model for STLF based on maximum entropy principle. Sun et al. [34,35] regarded STLF as a process of load information movement to tackle uncertainties in STLF, and then developed the minimization of Information Loss Based Hybrid STLF model to improve prediction accuracy, even though weather information is not involved separately, instead it was considered as a whole. It should be noted that, however, rich weather information available throughout various substations provides us a chance to further investigate the impact of meteorological factors on load demands. Thus, the key is to find an appropriate method of introduction of weather variables into STLF to characterize fully its influence on load demands.

Fisher information (FI) is well known for its ability to measure a system’s stability [32]. Unlike other approaches of system information measures, Fisher information provides a method that can monitor the system states’ changes and shifts by means of measuring the systems’ variables [36]. In fact, Fisher information has been applied to deriving fundamental equations of physics, thermodynamics, and population genetics [37,38]. More recently, Fisher information was widely applied to various areas, such as ecological systems [36,39], climate systems [40], power system fault detection [41], etc. The objective of this article is to demonstrate our beginning steps in the development of a methodology of introducing climatic factors into STLF models using Fisher information, and examine its effectiveness in practical application using a real data collected from a local utility company.

The remainder of the paper is organized as follows. Fisher information theory and its calculation are presented in Section 2. The description of introduction of meteorological factors and input variables selection based on FI for STLF models is given in Section 3. Different forecasting models are constructed with the proposed methodology in Section 4. Case study and discussion are presented in Section 5. Finally, the contributions with concluding remarks are reported in Section 6.

## 2. Methodology

The statistician Ronald Fisher (Fisher 1922), from a system’s stability point of view, developed a measure of indeterminacy, now called Fisher information. Fisher Information (FI) for a single measurement of one variable is calculated as follows [42]:

(1) $$I=\int^{}\frac{ds}{P\left(s\right)}{\left(\frac{dP\left(s\right)}{ds}\right)}^{2}$$

where *P*(*s*) is the probability density function (PDF), and *s* is a state variable.

A system’s stability is conceptually related to the repeatability of observations. Thus, for a system that is perfectly stable, repeated observations of the variables over time acquire the same values within the limits of measurement uncertainty. Hence, for perfect stability, the probability density function (*P*(*s*)) becomes a very sharp spike with a derivative d*P/*d*s* that is approaching infinity, and a Fisher information (see Equation (1)) is approaching infinity. For a system that is perfectly unstable, the opposite is true. Here, all observations acquire completely different and uncorrelated values for the variables, the likelihood of observing one value is the same as any other value, the probability density function (*P*(*s*), i.e., PDF) is flat, and the derivative d*P/*d*s* is approaching zero. In addition, when a system flips from a steady state to another, the derivate of the probability density function of the system shows an obvious transition. Real systems, however, exist between these two extremes of perfect stability and perfect instability and infinite and zero Fisher information. Hence, Fisher information is a theoretically sound measure of system stability.

In practical application, to reduce calculation errors due to dividing by small values of *P*(*s*), we replace the probability density function in Equation (1) with its amplitude, which is defined by *q*^2^(*s*) ≡ *P*(*s*). Equation (1) then becomes [43]:

(2) $$I=4\int ds{\left[\frac{dq\left(s\right)}{ds}\right]}^{2}$$

Note that in specific calculation, we do not know the concrete form of the continuous function *q*(*s*), but instead we have a finite number of samples *q_i_*. Thus, Fisher information (FI) is usually computed numerically. For that purpose, we shall replace the derivative by the numerical difference Δ*q* = *q*_i_ − *q*_i+1_ and Δ*s* = *s_i_* − *s_i_*_+1_, and correspondingly use the sum of finitely many terms to approximate the integral, which then leads to the following formula for calculating Fisher information approximately:

(3) $$I\approx 4\overset{n}{\underset{i=1}{\sum }}{\left[\frac{q_{i}-q_{i+1}}{s_{i}-s_{i+1}}\right]}^{2}\left(s_{i}-s_{i+1}\right)$$

In Equation (3), *s_i_* is an index denoting a particular state of the system, i.e., *s*_1_ is state 1, *s*_2_ is state 2, etc. Accordingly, *s_i_* − *s_i_*_+1_ = 1 and the final expression for computing Fisher information is:

(4) $$I\approx 4\sum {\left[q_{i}-q_{i+1}\right]}^{2}$$

The expression in Equation (4) will be used in all our Fisher information calculations afterwards.

### 2.1. FI Computation for the One-Dimensional Variables

The initial step in computing FI for the one-dimensional variables is to acquire an observed data series that characterize the state of the system over time. Then the time series data are divided into time windows by defining a parameter *w* denoting the size of the window. This parameter (*w*) is determined based on the amount of data available and the behavior of the system. From empirical studies, the *w* should contain at least eight time steps to ensure that one point in the window does not unduly influence the overall computation. A sequence of overlapping windows is then created to measure a system’s stability that may extend beyond the boundary of the window. This is achieved by moving the time window forward by a time increment (*δ*). The only rule regarding setting *δ* is ensuring that *w* > *δ*. The parameters *w* and *δ* denote the integration window size and window increment (in time steps) used to move through the data over time [43].

Suppose the measured time data serial is *D* = {d(1),…, d(*N*)}. We then introduce a series of sliding windows *W_m_* on *D* as follows

(5) $$W_{m}=\left\{d\left(k\right),\;\ldots d\left(k+w-1\right)\right\}$$

where *k* = 1 + *m* × *δ*, *w* ∈ *N* is the window size, *δ* ∈ *N* is the window increment, i.e., sliding factor, and *m* = 1, …, *M* with *M* = (*N* − *w*)/*δ* being the number of windows. Suppose all the elements in a sliding window *W_m_* can be divided into *I* intervals:

(6) $$W\left(m,w,\delta \right)=\overset{I}{\underset{i=1}{\cup }}Z_{i}$$

where

(7) $$Z_{i}=\left[S_{i-1},S_{i}\right),\;i=1,\;2,\;\ldots ,\;I$$

and

(8) $$S_{0}<S_{1}<S_{2}<\ldots <S_{I}$$

(9) $$S_{0}=\min\left[W\left(m,w,\delta \right)\right]=\min\left[\left\{d\left(k\right),\;k=1+m\times \delta ,\;\ldots ,w+m\times \delta \right\}\right]$$

(10) $$S_{I}=\max\left[W\left(m,w,\delta \right)\right]=\max\left[\left\{d\left(k\right),\;k=1+m\times \delta ,\;\ldots ,w+m\times \delta \right\}\right]$$

Then, the probability *P*(*Z_i_*) that the element d(*k*) falls into an interval *Z_i_* is:

(11) $$P\left(Z_{i}\right)=\frac{\mathrm{the}\;\mathrm{number}\;\mathrm{of}\;\mathrm{elements}\;\mathrm{falling}\;\mathrm{into}\;\mathrm{the}\;\mathrm{interval}\;Z_{i}}{\mathrm{the}\;\mathrm{total}\;\mathrm{number}\;\mathrm{of}\;\mathrm{elements}\;\mathrm{in}\;\mathrm{the}\;\mathrm{sliding}\;\mathrm{window}}$$

Next, the *q*(*Z_i_*) is calculated for each state ($q\left(Z_{i}\right)=\sqrt{p\left(Z_{i}\right)}$), and estimate FI value for each *W_m_*.

We summarize the procedures of calculating FI for the one-dimensional variables as follows: (i) Categorize a time series data into a sequence of time windows, overlapping each other. (ii) Divide each window into intervals with the same length by using the above method, and count the number of elements falling into each interval (iii) Construct a probability distribution function for the window. (iv) Calculate the Fisher information from the PDFs constructed in Step (iii) for each time window.

### 2.2. FI Computation for the Multidimensional Variables

Given a time series data with length *N* in *m*-dimensional state space are as follow.

{(*X*(*t*_1_), *X*(*t*_2_), …, *X*(*t_i_*), …, *X*(*t_N_*)}

where *X*(*t_i_*) = (*x*_1_(*t_i_*), *x*_2_(*t*_i_), …, *x*_m_(*t_i_*)) is a vector with m-dimensional components in the state space. Similarly, a series of overlapping windows each other are constructed on the above data series.

As stated above, the state of the system is defined by its state variables. The behavior of these state variables determines the stability of the system. Support the data points of representing the state variables within each window characterize a series of states for a system, and if $\left|X\left(t_{i}\right)-X\left(t_{j}\right)\right|\leq ∆X$ (∆X is a measurement’s allowed error), then both *X* (*t_i_*) and *X*(*t_j_*) are deemed as the same state. According to Chebyshev’s theorem [44], when ∆X takes 2δ (here, δ is the standard deviation of all the data points within a window), 75% of data points in this window will be categorized into the same state, no matter what the probability distribution is. Considering that each data point of the above window is an m-dimensional vector, an m-dimensional hyper-rectangle is constructed, and the lengths of the “*m*” sides of the hyper rectangles are determined by the uncertainties $2\delta_{1},2\delta_{2},\;\ldots ,\;2\delta_{m}$ in m-dimensional components respectively. (*δ*_1_, *δ*_2_, …, *δ_M_* are the standard deviation of data points for corresponding *m*-dimensional component within the window). More specifically, the first data point in chronological order within the first time window is taken as the center of the first state and a state hyper rectangle is constructed around it. All the points falling within the boundaries of the first state are counted or binned together, denoted as *Z*_1_, namely:

(12) $$Z_{1}=\left\{Z_{1}:\left(\left|x_{1}\left(t_{1}\right)-x_{1}\left(t_{j}\right)\right|\leq 2\delta_{1}\right)\;\&\left(\left|x_{2}\left(t_{1}\right)-x_{2}\left(t_{j}\right)\right|\leq 2\delta_{2}\right)\;\&\ldots \&\left(\left|x_{M}\left(t_{1}\right)-x_{M}\left(t_{j}\right)\right|\leq 2\delta_{M}\right)\right\},\;j=2,\;3,\ldots ,\;N.$$

The next uncounted point within the time window is selected, the second state is built around it, and the points are binned as before. Finally, this procedure is repeated until all the points are binned into states within the current time window, and the process moves to the next time window.

Suppose all the elements in a sliding data window can be put into *L* bins. Then, we have

(13) $$\mathrm{Length}\left(W\right)=\overset{L}{\underset{i=1}{\sum }}\mathrm{Length}\left(Z_{i}\right)$$

where Length(*W*) stands for the total number of data points contained in a window or bin of concern. That is, the total number of elements in a sliding window equals the sum of the subtotals contained in those bins.

We summarize the aforementioned procedures of the binning method: (i) Categorize a multidimensional time series into a sequence of time windows. (ii) In each window, convert data points into states by using the above method. (iii) In each window, construct a probability distribution function for possible states of the system. (iv) Compute the Fisher information from the PDFs constructed in Step (iii).

## 3. Methods of FI-Based Weather Variables Introduction and Feature Selection Process for STLF

Of all the weather factors, temperature is considered to have most influence on electrical demand. During high-temperature seasons, load demands increase sharply when the temperature rises. Particularly, a constantly high temperature has an accumulative effect on it, and a similar situation was found with the other weather variables. Accumulative effect of weather factors has a significant impact on STLF, and hence, it is taken into account in STLF [29].

FI is a measure of a system’s stability. The degree of the stability of the weather factors in previous period can be described quantitatively by measuring the FI value of past weather variables. In this way, current weather variables value is weighted using the FI value of weather factors in previous period, and then accumulative effect of weather factors on load demands is fully reflected. This approach is well in accordance with one’s general sense of weather variation caused by accumulative effect, that is, “steadily” low or high. It avoids identifying a cumulative effect coefficient regarding weather factors in conventional methods as well.

### 3.1. Methods of FI-Based Primitive Weather Variables Introduction into STLF Models

Fang et al. [28] investigated the relation between meteorological sensitive load and real-time temperature to examine accumulative effect of temperature on load demands. The result shows the effect comes from not only currently forecasted day, but previously several days. The former refers that a load demand at forecasted time point on the day was influenced by several previous time points before the time point within the day, especially for one previous time point, two previous time points, and three previous time points (in 1 h sampling time interval), while the latter refers that constantly high temperature within several previous days before the day would lead to grow unusually for the load demand. Specifically, these are one previous day and two previous days. Other weather variables show a similar result. Therefore, the sliding window for FI computation of a single weather variable, considering accumulation effect, consists of three sections as follows (in 15 min sampling time interval).

$$\left\{\left(X_{d,t}X_{d,t-1},\ldots ,X_{d,t-11}\right),\;\left(X_{d-1,t}X_{d-1,t-1},\ldots ,\;X_{d-1,t-7}\right),\;\left(X_{d-2,t}X_{d-2,t-1},\ldots ,\;X_{d-2,t-3}\right)\right\}$$

Note that the first subscript identifies day type, and the second one identifies time point. The subscript *d* and *t* represent currently forecasted day and time point, respectively, and the subscript (*d* − *i*) and (*t* − *j*) represent previous *i-*th day before the day and previous *j-*th time point before forecasted time point, respectively. As shown above, the first group is the sampling data points of the weather variables of 12 previous time points prior to the time point within the day. The second group is that of eight previous time points prior to the same time point on one previous day before the day, and the third group is that of four previous time points prior to the same time point on two previous days before the day. That is, the data window used for *FI* calculation comprises a total of 24 data points, which meets the principle that more sample real data should be selected when the distance regarding time between the sampled data points and the forecasted time point is much nearer and vice versa in handling those issues. This approach makes it more reasonable and comprehensive to depict accumulative impact of the weather factors on load demands.

### 3.2. Methods of FI-Based MCIs Introduction into STLF Models

MCIs can track more sensitively and describe more effectively load variation compared to the plain weather variables [19]. This is because one’s feeling of comfort is determined by an integrated effect mixing different weather factors such as temperature, humidity, wind speed, etc., together, while a single weather variable cannot characterize exactly one’s true feeling. In this context, several MCIs were introduced into STLF models, such as HI, THI, CIHB, etc.

Traditionally, the MCIs are defined by two or three weather variables to reflect an effect of interacting and inter coupling between them. One can image the effect is definitely associated with the stability of a system which is defined by corresponding weather variables, and the stability is measured by FI’s value of the multidimensional weather variables. The MCIs, similar to plain weather variables, have an accumulative effect on load demands, which lead to a time lag regarding one’s reaction to outside weather conditions. Accordingly, the effect should be considered when the STLF models are established, which is achieved by the weighted MCIs, namely, the MCIs multiplied by corresponding FI value.

The sliding window data serial for calculation multiple weather variables FI, considering accumulation effect, is constructed as follow.

$$\left\{\left(X_{d,t}X_{d,t-1},\ldots ,\;X_{d,t-11}\right),\;\left(X_{d-1,t}X_{d-1,t-1},\ldots ,\;X_{d-1,t-7}\right),\;\left(X_{d-2,t}X_{d-2,t-1},\ldots ,X_{d-2,t-3}\right)\right\}$$

As displayed above, it consists of three parts similar to the single weather variables in Section 3.1. It is worth noting that each element in the above window, e.g., *X_d,t_* = (*x_d,t_*(1), *x_d,t_*(2),…, *x_d,t_*(M)), is a vector with *m*-dimensional components in the weather state space. Data selection for the sliding window and the symbols’ meanings are the same as Section 3.1.

### 3.3. FI-Based Feature Selection Process for STLF Models

It is also relevant to notice that the forecasting engine is only a part of an accurate forecasting model and other processes such as feature selection (FS) are very important as well [45]. FS is commonly applied to identifying the most significant input variables influencing STLF’s prediction accuracy. The variables selection is very crucial for artificial intelligence (AI) models training. Input variables, through selecting reasonably, can improve STLF’s prediction accuracy and reduce models’ training costs. Therefore, how to choose the most important factors impacting on load demands from massive historical data to yield a set of effective and sufficient input variables is a challenging task. Drezga et al. [46] proposed a phase-space embedding method to identify input variables for NN models, but it is only able to find out historical loads that have the most impact on the future forecasted time point, and has limited effect for the weather variables. Gao et al. [47] applied orthogonal least square (OLS) approach to select feature for NN models in STLF. It, however, require to identify a measurement’s allowed error in advance. Liu et al. [48] adopted Relief algorithm to address FS matter for STLF, but computation process is more complicated.

Essentially, Fisher Information is the inverse of the Shannon entropy; Where Shannon entropy measures the degrees of disorder or indeterminacy of the system, while Fisher Information measures the degrees of order or determinacy of the system. For a certain thing, the more deterministic it is, the more it outputs information, the more regular its structure is, and the larger its FI value is. By means of this property, the importance degree of all the input variables in STLF can be identified. The concrete implementation process is as follow.

Suppose a historical daily average load consumption dataset with length *m* is used to analyze the impact of *n* factors on load, and the following matrix for the analysis is then constructed:

$$X={\left(x_{ij}\right)}_{m\times n}=\left(\begin{array}{ccc} x_{11} & \ldots & x_{1n}\\ \vdots & \ddots & \vdots \\ x_{m1} & \ldots & x_{mn} \end{array}\right)$$

To avoid the effect of different dimension, each element in the matrix is normalized to [0, 1] to acquire a normalized matrix $R={\left(r_{ij}\right)}_{m\times n}$. FI value regarding per column corresponding to a factor is calculated, then is normalized to [0, 1] once again. The weight of the *j-*th influencing factor is computed as follow.

(14) $$W_{j}=\frac{1-{\mathrm{FI}}_{j}}{n-\sum_{j=1}^{n}{\mathrm{FI}}_{j}}$$

As can be seen in Equation (14), when the *j-*th factor all takes the same value in the *j-*th column, the factor’s FI value takes the maximum 1, while its weight *W_j_* takes the minimum 0, which signifies the factor cannot provide any useful information for STLF. The factor should be ignored or deleted from the above influencing factors. Conversely, when the *j-*th factor takes completely different and uncorrelated values in the *j-*th column, the factor’s FI value takes the minimum, while its weight *W_j_* takes the maximum, which signifies the factor can provide useful information and much attention should be paid to the factor in STLF. As a result, the *j-*th factor’s value is weighted using its weight *w_j_* above to acquire a final value as the *j-*th input variable of STLF models. All the rest of the input variables are determined in the same way. FI-based feature selection process (FSP) can reduce the redundant information and improve the prediction accuracy and speed.

## 4. Load Forecasting Models

Over the past few decades, using weather variables as an integral part of forecasting process has been the most populous. It is common approach among neural network (NN) ones. Of all these methods using weather variables, backpropagation (BP) NN is one of the most widely applied means. The reason for this is that it can approximate numerically any continuous function to the desired accuracy. In addition, it is data-driven methods, in the sense, it is not necessary for the researchers to postulate tentative models and then estimate their parameters [49]. The most common BP structure has three layers with a sigmoid transfer function for the activation function in the hidden layer and a linear transfer function in the output layer. The possible drawbacks for the method are, first, no theoretical approach can be applied to calculating the appropriate number of hidden layer nodes [50]. Secondly, it is prone to overfit sample data because of the large number of parameters that must be estimated [49]. In consideration of this, it is adopted to demonstrate the effectiveness of FI-based weather variables introduction and feature selection process for STLF, although it should be noted that the choice of modeling technique is not central to this paper. 

### 4.1. Forecasting Model with Primitive Weather Variables

Previous load forecasting methods mainly consider characteristics of daily meteorological factors, yet out of consideration is the influence of meteorological factors on load at different time points over a day. For depicting pointwise influence of meteorological factors on the load by means of primitive weather variables, a BP NN-based STLF model with 19 inputs variables is built [31]. The model consists of three layers, namely the input layer, the hidden layer, and the output layer, and the output layer has only one variable, i.e., the forecasted load at this point. Temperature, humidity, and load, both at different points in the same day and at the same point in different days, and others, are defined as input variables for the model. All the input variables are shown in Table 1.

Nineteen variables are applied to the input neurons of BP neural network algorithm to examine the impact of real-time weather factors on load. This model is adopted as the benchmark method and denoted as Model I, for comparing with the following methods based on FI, in this paper.

### 4.2. Forecasting Model with Primitive MCIs

For a long time, MCIs have played a significant role in introducing weather variables into STLF models, and tey exhibit a comprehensive effect for outside weather conditions compared to the primitive weather variables. Therefore, it is imperative to construct a BP NN-based STLF model with primitive MCIs in terms of real-time MCI influence on load, which is achieved by using an MCI (specifically, THI) instead of both temperature and humidity in Model I. The model is presented in Table 2.

This model with 13 input variables is denoted as Model II to demonstrate the impact of real-time MCI on load, and also to compare it with Model I above.

### 4.3. Forecasting Model with Weighted Weather Variables Based on FI

Using the aforementioned Model I as a basis, temperature and humidity are renewably introduced the model by means of FI-based approach, and then, the input variables are reduced to 11. They are displayed in Table 3.

This model with 11 input variables is denoted as Model III. The model can describe well accumulative effect of both temperature and humidity on load demands by means of the proposed method. In addition, it offsets the time differences between load variation and weather conditions, which makes the variation of load with weather conditions to exhibit a real-timely effect. 

### 4.4. Forecasting Model with Weighted MCIs Based on FI

THI is one of the most widely applied MCIs because of its practicability. As a result, the THI is utilized for demonstrating the performance of the method of weighted MCIs introduction into STLF models. The THI is the temperature and humidity index, which can be calculated with the following formula [19]:

(15) $$\mathrm{THI}=T+\frac{1450.8\left(T+235\right)}{4030-\left(T+235\right)\ln R}-43.4$$

where *T* and *R* are temperature (in degrees Celsius) and relative humidity (integer percentage), respectively. The THI value computed multiplies by two-dimensional FI value of both temperature and relative humidity as a weighted THI (WTHI), as stated in Section 3.2, and is introduced BP NN model. In this manner, the input variables are reduced to nine. They are described in Table 4.

This model with nine input variables is denoted as Model IV. The model is established, using the proposed method, to characterize the accumulation impact of the MCIs on load point by point. It is supposed to be capable of further improving the accuracy and speed of STLF.

## 5. Case Study and Discussion

Here is a case study designed to demonstrate the performance of the proposed methods. The historical load and meteorological dataset are collected from Zhenjiang utility company and Zhenjiang Weather Bureau, respectively, Jiangsu Province, in southeast China. The load dataset used for the simulation were sampled every 15 min, from 1 August 2016, to 31 August 2016. The weather dataset collected in corresponding period were sampled every 1 h, and then interpolated to produce corresponding sampling interval dataset. Therefore, 96 points of load and weather data (involving temperature, humidity, and wind speed), day type, THI are included in the data of each day. The simulation is implemented with MATLAB 2015b (MathWorks, Natick, MA, USA).

### 5.1. Comparison of the Correlation between the Load Demands and the Weather Variables

The comparison of the correlation among daily average load and mean temperature before and after weighted with the proposed method is performed. Note that both daily average load and corresponding mean temperature are taken by Per-Unit Value.

**Figure 1:**
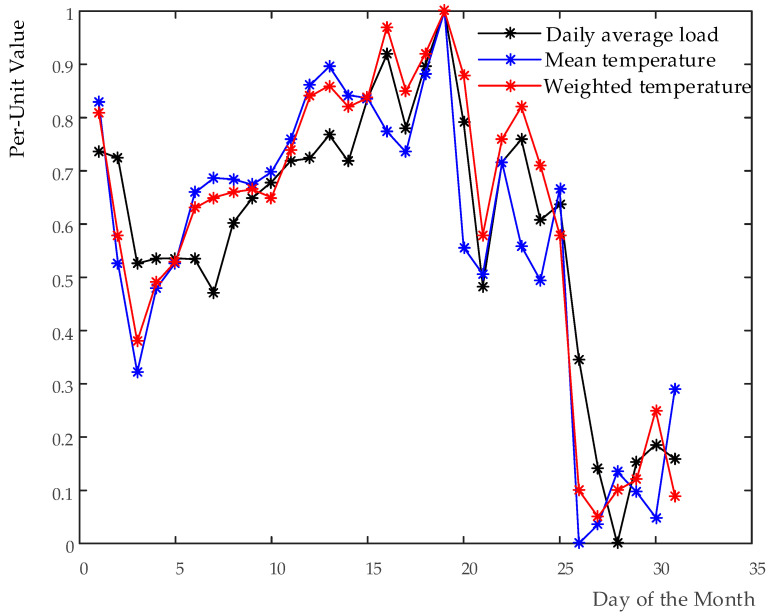
Comparison of the curves of daily average load and mean temperature before and after weighted.

As shown in Figure 1, the daily average load has an evolution trend similar to a primitive temperature as a whole, which matches to the anticipation. In contrast, the weighted temperature (WT) has a much better characterization for the evolution rules of the load. It can be observed that the load stays in rising stage when the WT rises, while the load is in decreasing stage when the WT falls. Moreover, the peak and valley values of the curve of the load are well corresponding to that of the WT, which show a much stronger correlation between the load and the WT. It is worth noting that the variations of the load of certain days are not fully consistent with that of the temperature of corresponding days. This is because temperature is not an only influencing factor on load changes. 

**Figure 2:**
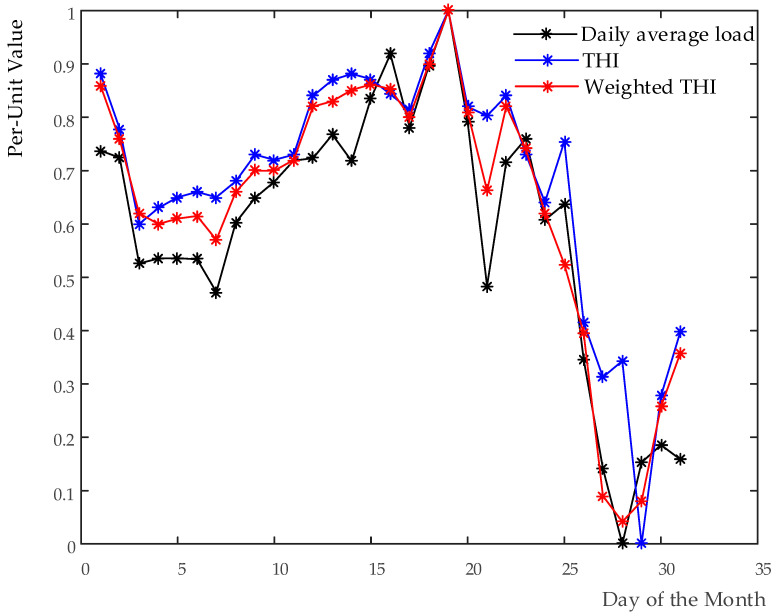
Comparison of the curves of daily average load and THI before and after weighted.

Figure 2 presents the comparison of the correlation among the load and THI before and after weighted. As displayed in the figure, a similar result is found with the THI. 

The values of the measure of the correlations among the aforementioned variables are calculated based on the following formula:

(16) $$r\left(x,y\right)=\frac{\sum_{t=1}^{n}\left(x_{t}-\bar{x}\right)\left(y_{t}-\bar{y}\right)}{\sqrt{\sum_{i=1}^{n}{\left(x_{t}-\bar{x}\right)}^{2}}\sqrt{\sum_{i=1}^{n}{\left(y_{t}-\bar{y}\right)}^{2}}}$$

*r*(*x*, *y*) represents the correlation coefficient between the variable *x* and *y*; *x_t_* and *y_t_* refer to the value of *x* and *y* at *t* time point, respectively; $\bar{x}\;\mathrm{and}\;\bar{y}$ identify the mean value of *x* and *y*, respectively; and *n* is the number of sampling time points. The measured values are illustrated in Table 5. 

It can be seen in Table 5 that, using the proposed method, the correlation coefficient between the load and the temperature increases from 0.88 to 0.94, while that of both the load and the THI grows from 0.91 to 0.96. Obviously, the correlations among them are improved. All these analyses confirm the feasibility of the proposed approach, which lay a foundation for the following discussion.

### 5.2. Comparison of the Prediction Effect of Different Forecasting Models

Based on the aforementioned load forecasting models and the analysis of feature selection in Section 4, a load in term of every 15 min on 10 August 2016 is forecasted point by point. The mean absolute percentage error (MAPE) $e_{\mathrm{MAPE}}$ and the margins of forecasting error *A_C_* are adopted as the criteria of evaluating the performances of load forecasting models, respectively:

(17) $$e_{\mathrm{MAPE}}=\frac{1}{n}\overset{n}{\underset{i=1}{\sum }}\left|\frac{A\left(i\right)-F\left(i\right)}{A\left(i\right)}\right|\times 100\%$$

(18) $$A_{C}=\sqrt{\frac{1}{n}\overset{n}{\underset{i=1}{\sum }}{\left(\frac{A\left(i\right)-F\left(i\right)}{A\left(i\right)}\right)}^{2}}\times 100\%$$

where *n* is the number of forecasting time points; *F*(*i*) is the forecasted load at time point *i*; *A*(*i*) is the actual load at corresponding time point; and *A_C_* represents the average prediction variance of load forecasting in 96 points.

**Figure 3:**
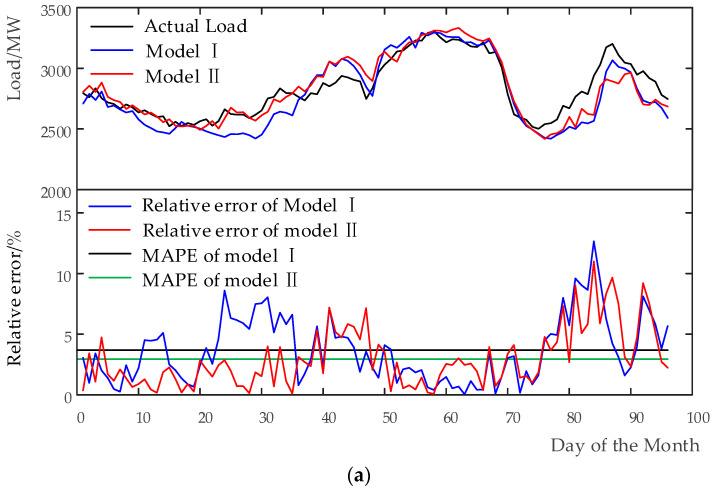

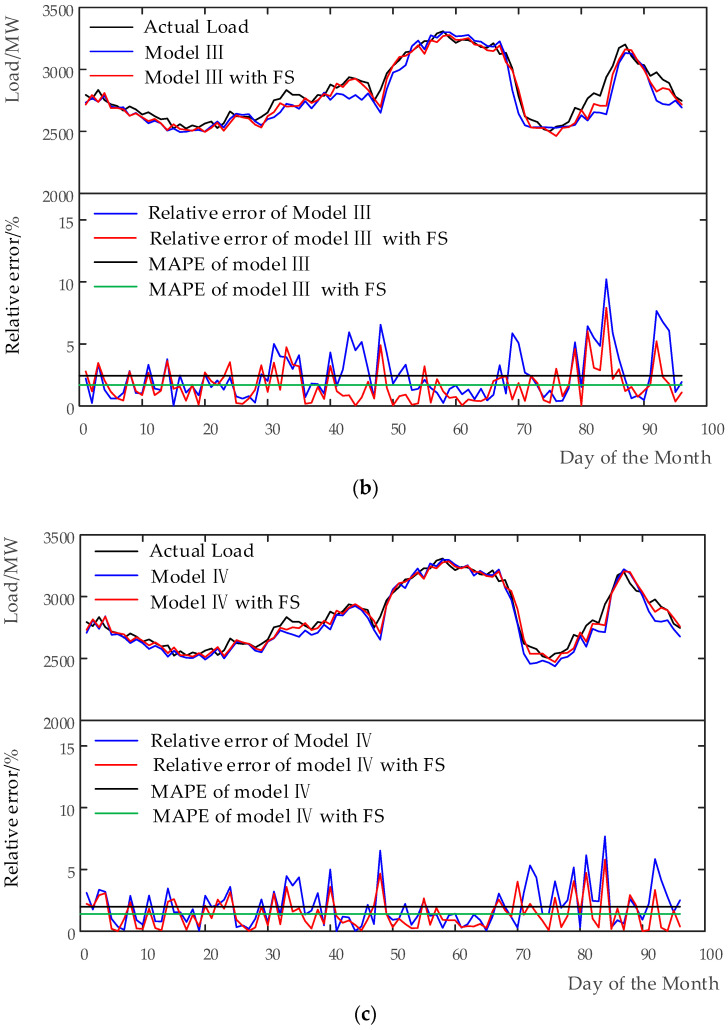
(**a**) Forecasting output and error analysis of both Model I and Model II on 10 August 2016; (**b**) forecasting output and error analysis of Model III before and after FS on 10 August 2016; and (**c**) forecasting output and error analysis of Model IV before and after FS on 10 August 2016.

Figure 3 exhibits the prediction results, the relative error curve, and MAPE for different forecasting models. Figure 3a depicts the actual load demand and the forecasted load demand using the benchmark model i.e., Model I and Model II. The relative error curves is presented as well. As shown in the figure, the forecasted load demand with Model II better matches the actual one compared to Model I, and the prediction performances with Model II is better than Model I in term of both relative error and MAPE. Figure 3b displays those of Model III before and after FS. From this figure, it can be observed that the forecasted load demand obtained using Model III is better than both Model I and Model II, and Model III with FS is better than the one without FS, which implies FS has an impact on the accuracy of STLF. Overall, the amplitude of the relative error curves plotted in Figure 3b are lower and more homogeneous than both Model I and Model II, and the MAPE obtained in Figure 3b) is less compared to Figure 3a. Figure 3c characterizes the actual load demand and forecasted load demands of Model IV before and after FS. It can be seen clearly that the prediction results shown in Figure 3c is much better compared to Figure 3a,b. Particularly, Model IV with FS displays the best prediction performance among the models, and the forecasted load demand with Model IV through FS closely matches the actual load demand. Correspondingly, the amplitude of the relative error curves of the model is much smaller and more homogeneous than others, and its MAPE is the smallest one in all the models. In summary, the proposed models can achieve better forecasting results in comparison with both Model I and Model II in Figure 3. 

The relative error of hourly load forecasting results among different models is listed in Table 6. Subsequently, $e_{\mathrm{MAPE}}$ and A_C_ are presented to further assess their respective performance. As can be seen in Table 6, a smaller $e_{\mathrm{MAPE}}$ of load forecasting performance can be clearly observed in the proposed models when compared with both Model I and Model II. The $e_{\mathrm{MAPE}}$ of Model II decreased from 3.68% to 2.97%, and the A_C_ reduced from 4.54% to 3.84%. Those of Model III decreased from 3.68% to 2.43%, and from 4.54% to 3.13%, respectively. Those of Model IV decreased from 3.68% to 1.98% and from 4.54% to 2.55%, respectively. Those of Model III with FS decreased from 3.68% to 1.69% and from 4.54% to 2.22%, respectively. Those of Model IV with FS decreased from 3.68% to 1.39% and from 4.54% to 1.86%, respectively. overall, the accuracy of the load prediction is improved step by step from Model I to Model IV with FS, which is in accordance with the anticipation.

## 6. Conclusions

The principal contributions in this paper are to propose a robust methodology as a practical means of both introducing weather factors into STLF models and selecting its input variables, and comparing it with traditional methods. 

Weather information is an important factor in load forecasting models. How to effectively introduce meteorological variables and reasonably select feature for STLF models has always been a topic of interest in this area. When looked at from an information point of view, FI can be interpreted as the amount of information that can be extracted from a set of measurements. From this point, the amount of weather information in previous period can be measured from past weather variables by FI. In this way, current weather variables value is weighted using the FI value of weather information in previous period, and then accumulative effect of weather factors on load demands is fully reflected. The same applies to FS in STLF models.

The advantages of the proposed method are easy to understand and convenient to apply in practical load forecasting. As demonstrated above, the method overcomes the main drawback of traditional method in term of considering accumulative effect of both the plain weather variables and the MCIs on load, and displaying a real-time effect on the load demands. In addition, the proposed FS approach reduces the redundant information, saves model training time and improves STLF’s prediction speed. Different forecasting models with the proposed approach are established and implemented, based on the case study of Zhenjiang City in southeast China, to assess the performance of the methodology. The simulation results obtained show the usefulness of the proposed methodology, which can be used as a unified method for weather variables introduction and feature selection for STLF models.

## Figures and Tables

**Table 1:** The description of input variables.

Input Variables	Description
1–2	day type and time point for a forecasted load.
3–4	load at one and four previous time points before the time point on the day.
5–10	temperature and humidity at the time point, one and four previous time points on the day.
11–13	load at the time point, one and four previous time points on the same day in last week.
14–19	temperature and humidity at the time point, one and four previous time points on the same day in last week.

**Table 2:** The description of input variables.

Input Variables	Description
1–2	day type and time point for a forecasted load.
3–4	load at one and four previous time points before the time point on the day.
5–7	THI at the time point, one and four previous time points on the day.
8–10	load at the time point, one and four previous time points on the same day in last week.
11–13	THI at the time point, one and four previous time points on the same day in last week.

**Table 3:** The description of input variables.

Input Variables	Description
1–2	day type and time point for a forecasted load.
3–4	load at one and four previous time points before the time point on the day.
5–6	weighted temperature and humidity at the time point on the day.
7–9	load at the time point, one and four previous time points on the same day in last week.
10–11	weighted temperature and humidity at the time point on the same day in last week.

**Table 4:** The description of input variables.

Input Variables	Description
1–2	day type and time point for a forecasted load.
3–4	load at one and four previous time points before the time point on the day.
5	weighted THI at the time point on the day.
6–8	load at the time point, one and four previous time points on the same day in last week.
9	weighted THI at the time point on the same day in last week.

**Table 5:** The similarity among different curves.

Item	Load	T	WT	THI	WTHI
Load	1.00	0.88	0.94	0.91	0.96
T	—	1.00	0.92	0.90	0.89
WT	—	—	1.00	0.91	0.93
THI	—	—	—	1.00	0.95
WTHI	—	—	—	—	1.00

WT: the weighted temperature; THI: Temperature–humidity index; WTHI: a weighted THI.

**Table 6:** Comparison of hourly load forecasting output (FS: feature selection).

Hour	Actual Load (MW)	Model I (%)	Model II (%)	Model III (%)	Model III with FS (%)	Model IV (%)	Model IV with FS (%)
00:00	2794.70	3.04	0.37	2.20	2.78	3.11	2.22
01:00	2718.78	1.38	1.69	0.59	1.14	1.01	0.20
02:00	2677.85	1.08	0.64	1.03	1.20	0.91	0.23
03:00	2598.20	4.54	0.17	1.27	1.22	0.80	0.07
04:00	2524.42	1.35	0.19	1.07	0.42	0.75	0.11
05:00	2580.44	3.85	2.13	1.51	1.99	1.97	1.05
06:00	2624.22	6.32	1.99	0.76	0.23	0.32	0.92
07:00	2617.43	7.48	1.85	2.55	3.27	2.57	1.98
08:00	2834.88	6.76	3.94	3.93	4.73	4.46	3.60
09:00	2735.86	1.72	2.71	1.80	0.25	1.67	0.22
10:00	2852.19	7.19	7.19	1.61	1.18	0.01	1.22
11:00	2904.61	3.92	5.61	5.15	0.68	0.39	0.06
12:00	2968.99	1.39	4.13	4.23	1.47	1.38	1.40
13:00	3148.01	2.10	0.54	1.27	0.09	0.52	0.24
14:00	3291.50	0.61	0.18	1.05	2.15	1.35	1.88
15:00	3240.10	0.53	2.45	0.93	0.05	0.29	0.33
16:00	3175.92	0.41	1.93	0.44	0.65	0.06	0.27
17:00	3009.97	1.44	1.44	5.86	0.51	1.14	1.18
18:00	2575.66	1.95	1.55	1.62	1.84	4.34	1.48
19:00	2548.53	5.02	3.67	0.41	0.77	1.89	0.33
20:00	2766.07	9.59	9.02	6.43	6.01	6.15	4.72
21:00	3028.17	9.53	5.89	5.88	2.16	0.31	0.17
22:00	3047.15	1.60	3.13	0.84	0.73	1.88	2.05
23:00	2918.87	6.99	7.56	6.77	2.34	4.15	0.30
e_MAPE_ (%)	_	3.68	2.97	2.43	1.69	1.98	1.39
*A_C_* (%)	_	4.54	3.84	3.13	2.22	2.55	1.86
